# Preoperative Albumin, Transferrin, and Total Lymphocyte Count as Risk Markers for Postoperative Complications After Total Joint Arthroplasty: A Systematic Review

**DOI:** 10.5435/JAAOSGlobal-D-19-00057

**Published:** 2020-09-04

**Authors:** Chukwuemeka Mbagwu, Matthew Sloan, Alexander L. Neuwirth, Ryan S. Charette, Keith D. Baldwin, Atul F. Kamath, Bonnie Simpson Mason, Charles L. Nelson

**Affiliations:** From the Howard University College of Medicine (Dr. Mbagwu), Washington, DC; the Mount Sinai Hospital (Dr. Mbagwu), New York City, NY; the Department of Orthopaedic Surgery (Dr. Sloan, Dr. Charette, Dr. Kamath, Dr. Nelson), University of Pennsylvania; the Department of Surgery (Dr. Sloan, Dr. Charette, Dr. Nelson), University of Pennsylvania, Philadelphia, PA; the Department of Orthopaedic Surgery (Dr. Neuwirth), Columbia University Medical Center, New York City, NY; the Division of Orthopaedic Surgery (Dr. Baldwin), Department of Surgery, Children's Hospital of Philadelphia, Philadelphia, PA; the Orthopaedic and Rheumatologic Institute (Dr. Kamath), Cleveland Clinic, Cleveland, OH; the Nth Dimensions, Chicago, IL, (Dr. Mason); the University of Louisville School of Medicine (Dr. Mason), Louisville, KY; and the University of Texas Medical Branch (Dr. Mason), Galveston, TX.

## Abstract

**Introduction:**

The purpose of this systematic review is to identify whether poor nutrition, as defined by the more commonly used markers of low albumin, low transferrin, or low total lymphocyte count (TLC), leads to more postoperative complications. We hypothesized that it may be possible to identify the levels of these laboratory values at which point total joint arthroplasty (TJA) may be ill advised. To this end, we analyzed the available literature regarding links between these three variables on postoperative complications after TJA.

**Methods:**

This systematic review was done in two parts: (1) In the first part, we reviewed the most commonly used malnutrition marker, albumin. (2) In the second part, we reviewed TLC and transferrin. We accessed PubMed, EMBASE, and Cochrane Library using relevant keywords to this study. The biostatistics were visualized using a random-effects forest plot. We compared data from all articles with sufficient data on patients with complications (ie, cases) and patients without complications (ie, noncases) among the two groups, malnourished and normal nutrition, from albumin, transferrin, and TLC data.

**Results:**

A meta-analysis of seven large-scale articles detailing the complications of albumin led to an all-cause relative risk increase of 1.93 when operating with hypoalbuminemia. This means that in the studies detailed enough to incorporate in this pooled analysis, operating on elective TJAs with low albumin is associated with a 93% increase in all measured complications. In the largest studies, analysis of transferrin levels for the most common complications revealed a relative risk increase of 2.52 when operating on patients with low transferrin levels. There were not enough subjects to do a biostatistical analysis in articles using TLC as the definition of malnutrition.

**Conclusion:**

The focus is on the trends rather than absolutes. As shown in Table 1, whether the albumin cutoff for albumin was 3.0 g/dL, 3.5 g/dL, or 3.9 g/dL, the trend remains the same. Because low albumin before TJAs tends to increase complications, it is recommended to incorporate albumin levels in preoperative workups. Many patients with hip and knee arthritis undergo months of conservative management (eg, physical therapy and corticosteroid injections) before considering surgery, and it would be wise to optimize their nutritional status in this period to minimize the risk of perioperative complications. The physician should use these data to provide informed consent of the increased risk to patients planning to undergo TJAs with elevated malnutrition markers. Because this research is retrospective in nature, albumin should be studied prospectively in hypoalbuminemic and normoalbuminemic patients and their postoperative outcomes should be measured. Regarding transferrin and TLC, future research should help elucidate their predictive value and determine the value of preoperatively optimizing them and their effect in mitigating postoperative complications.

Poor nutritional status before total joint arthroplasty (TJA) can lead to perioperative complications such as wound complications,^1^ infection,^2-4^ or even mortality.^5,6^ Despite general agreement with this statement, the primary metric by which malnutrition is demonstrated is less clear. Several laboratory markers have been suggested as indicative of poor nutritional status. Among these, the more commonly used markers are albumin,^7^ transferrin,^8^ and total lymphocyte count (TLC).^1,9^

The purpose of this systematic review is to identify whether poor nutrition, as defined by low albumin, low transferrin, or low TLC, leads to more postoperative complications. We hypothesized that it may be possible to identify the levels of these laboratory values at which point TJA may be ill advised. To this end, we analyzed the available literature regarding links between these three variables on postoperative complications after TJA.

## Methods

This systematic review was done in two parts:

(1) In the first part, we reviewed the most commonly used malnutrition marker, albumin.
(2) In the second part, we reviewed TLC and transferrin.

During the first part, we accessed PubMed, EMBASE, and Cochrane Library using search phrases with the following keywords: “albumin,” “pre-albumin,” “prealbumin,” “total joint arthroplasty,” “total joint replacement,” “total hip arthroplasty,” “total hip replacement,” “total knee arthroplasty,” “total knee replacement,” “infection,” “complication,” “readmission,” “readmit, “postoperative,” and “perioperative.” This yielded 312 results in PubMed, 15 results in EMBASE, and 88 results in Cochrane Library. These titles were reviewed by independent reviewers. Pertinent English-language articles were considered for inclusion in the final review, as were all duplicates. Screening of the initial titles yielded 23 PubMed articles, five EMBASE articles, and one Cochrane Library article. Of these 29 titles, three were duplicates. One additional article was identified during the full-text article retrieval and was included in the systematic review, yielding a total of 30 articles related to albumin. We reviewed the following items in the 29 obtainable articles: year of publication, primary outcome, albumin cutoff level used to define hypoalbuminemia, patient source, sample size, follow-up duration, study design, complications evaluated, and relative risk (RR) of complications among patients with hypoalbuminemia. The resulting studies are listed in Tables 1–4 and Table 9.

**Table 1:** Demographics of Albumin Studies

Author Name	Study Population	Year	Mean Follow-up Time	Study Type	Hypoalbuminemic Cutoff	Normal Albumin Patient (Total)	Low Albumin Patient (Total)
Alfargieny et al^2^	THA and TKA	2015	6 mo	R	Not given	Not given	5
Bohl et al^3^	THA and TKA	2016	30 d	R	<3.5 g/dL	47,639	1964
Bohl et al^10^	THA and TKA	2016	30 d	R	<3.5 g/dL	3762	755
Courtney et al^11^	THA and TKA	2016	6 mo	R	<3.5 g/dL	587	83
Cross et al^7^	Not applicable (N/A)	2014	N/A	N/A	N/A	N/A	N/A
Fu et al^12^	THA	2016	30 d	R	<3.5 g/dL	19,465	745
Fu et al^5^	TKA	2017	30 d	R	<3.5 g/dL	33,400	1400
Gherini et al^8^	THA	Not given	Not given	P	Not given	Not given	Not given
Greene et al^1^	THA and TKA	1991	1 year	R	<3.5 g/dL	211	6
Gunningberg et al^13^	THA, TKA, and coronary artery bypass	2008	30 d	P	<35 g/L	51	4
Huang et al^14^	THA and TKA	2013	12 mo	P	Albumin <3.5 mg/dL OR transferrin <200 mg/dL	Not given	Not given
Kamath et al^15^	THA and TKA	2016	Not given	P	<3.5 g/dL	913	185
Kamath et al^6^	Revision TKA	2017	30 d	R	<3.5 g/dL	3838	713
Kim et al^16^	TKA	2016	4.2 y	R	<3.0 g/dL	839	470
Lavernia et al^17^	THA and TKA	1999	Not given	P	<or = 34 g/L	97	22
Marín et al^9^	THA and TKA	2002	Not given	P	<3.5 g/dL	152	18
Mednick et al^18^	THA	2014	30 d	R	Not given	Not given	Not given
Morey et al^19^	TKA	2016	1 year	R	<3.5 g/dL	2956	213
Nelson et al^4^	TKA	2015	30 d	R	<3.5 g/dL	35,573	1570
Nelson et al^20^	THA	2019	30 d	R	<3.5 g/dL	23,409	1177
Nicholson et al^21^	THA	2012	Not given	R	Albumin <3.5 g/dL AND TLC <1.50 cells/m	64	26
Nussenbaum et al^22^	THA and TKA	2018	2 y	R	Not given	Not given	Not given
Rai et al^23^	THA and TKA	2002	Not given	R	Albumin <3.5 g/dL OR serum transferrin <200 mg/dL, OR TLC <1500 cells/mm3	Not given	Not given
Savio et al^24^	THA	1996	1.8 y	R	Albumin <3.5 g/dL, then changed to albumin <3.9 g/dL is normal after the researchers analyzed the data	51	35
Walls et al^25^	THA	2015	30 d	R	<3.5 g/dL	23,116	1122
Yi et al^26^	Revision THA	2014	90 d	R	<3.5 g/dL	Not given	Not given

N/A = not applicable; P = prospective; R = retrospective; THA = total hip arthroplasty; TKA = total knee arthroplasty; TLC = total lymphocyte count

Table 1 explains the different demographics of each of the articles of albumin, minus the outcomes and conclusions, which are listed in Table 2.

**Table 2:** Outcomes and Conclusions of Albumin Studies

Author Name	Outcomes	Conclusions
Alfargieny et al^2^	SSI	Perioperative albumin was a notable risk factor for SSI
Bohl et al^3^	30-day postoperative outcomes—wound dehiscence, deep vein thrombosis, and other	Patients with hypoalbuminemia had a higher risk of SSI, pneumonia, extended length of stay, and readmission
Bohl et al^10^	Aseptic indications for revision arthroplasty, septic indications for revision arthroplasty, and PJI	Patients with hypoalbuminemia were three times as likely to have septic indication for revision arthroplasty as compared to patients with normal albumin levels. For those with aseptic indications for revision arthroplasty, patients with hypoalbuminemia had a risk of developing a PJI twice as great as those with normal albumin levels.
Courtney et al^11^	Postoperative complications: cardiac, pulmonary, and other	Hypoalbuminemic patients were more likely to have a postoperative complication. Morbidly obese patients were more likely to be hypoalbuminemic than nonmorbidly obese patients. When comparing hypoalbuminemic morbidly obese patients with hypoalbuminemic nonmorbidly obese patients, no differences were observed in complication rates. When comparing morbidly obese patients with normal albumin to nonmorbidly obese patients, no differences were observed in complication rates.
Cross et al^7^	N/A	N/A
Fu et al^12^	Postoperative complications: cardiac (ie, myocardial infarction or cardiac arrest), septic (ie, sepsis or septic shock), and other	Malnutrition incidence increased markedly from obese I to obese III patients and was a stronger and more consistent predictor of complications after THA than was obesity.
Fu et al^5^	Postoperative complications: wound (ie, superficial infection, deep SSI, organ space surgical site infection, or wound dehiscence) and other	Hypoalbuminemia was a more consistent independent predictor of complications after TKA than was obesity.
Gherini et al^8^	Delayed wound healing	Only preoperative serum transferrin levels showed notable value in predicting which patients would have delayed wound healing. None of the other serologic variables, including serum albumin and TLC, proved to be a predictor of delayed wound healing.
Greene et al^1^	Persistent serous drainage and wound dehiscence	Low albumin and low transferrin, independently or concurrently, were associated with more postoperative complications.
Gunningberg et al^13^	Surgical wound infection	Low preoperative S-albumin was identified as the only notable predictor for surgical wound infection.
Huang et al^14^	Complications: cardiovascular, neurovascular, and other	The incidence of complications was higher in malnourished patients than in nonmalnourished patients, regardless of whether they were obese. Renal complications were the most common complication experienced by malnourished patients and occurred at markedly higher rates than for nonmalnourished patients. Age was not a notable factor in developing malnutrition, but the incidence increased steadily through age 70.
Kamath et al^15^	Unplanned postoperative intensive care unit admission	Patients with low albumin had a higher risk of unplanned postoperative intensive care unit admission.
Kamath et al^6^	Mortality, superficial wound infection, and other	Patients in the low serum albumin group were statistically more likely to develop deep SSI, organ space SSI, and other complications.
Kim et al^16^	Incidence of acute kidney injury, hospital stay, and overall mortality	Low albumin within two postoperative days was an independent risk factor for acute kidney injury and increased length of hospital stay in patients undergoing TKA.
Lavernia et al^17^	Complications, resource consumption, length of stay, and number of in-hospital medical or surgical consults obtained	Patients with low albumin levels had higher charges, higher severity of illness, and longer length of stay.
Marín et al^9^	Delayed wound healing	Preoperative lymphocyte count of less than 1500 cells/mm^3^ was associated with a three times greater frequency of healing complications, whereas preoperative serum albumin and transferrin levels had no notable predictive value.
Mednick et al^18^	Readmission	The risk of readmission after THA increased with growing preoperative comorbidity burden. It specifically increased in patients with a body mass index of greater than or equal to 40 kg/m^2^, a history of corticosteroid use, and low preoperative serum albumin and in patients with postoperative SSI, a thromboembolic event, and sepsis.
Morey et al^19^	Wound complications (ie, drainage, hemarthrosis, skin necrosis, and dehiscence) or PJI	Findings called into question the values of serum albumin level and TLC as a surrogate of malnutrition for predicting wound complications after TKA.
Nelson et al^4^	Mortality, superficial wound infection, and other	Morbid obesity was not independently associated with most perioperative complications measured by the ACS-NSQIP and was associated only with increases in progressive renal insufficiency, superficial SSI, and sepsis among the 21 perioperative variables measured. Low serum albumin was associated with increased mortality and multiple additional major perioperative complications after TKA. Low serum albumin, more so than morbid obesity, was associated with major perioperative complications.
Nelson et al^20^	Major complications, wound infections, and systemic infections	ORs increased or trended higher for all complications for albumin levels under 3.0 g/dL
Nicholson et al^21^	Length of stay, intraoperative complications, and postoperative complications	The rate of malnourishment was marked higher in patients having trauma-related surgery than in those having elective surgery. Malnourished patients were at greater risk of prolonged hospital stay.
Nussenbaum et al^22^	SSI, return to operating room, and other	The investigators saw a statistically significant decrease in both SSI and total complications after the implementation of preoperative screening criteria for elective TKA and THA. No single criterion was found to individually predict the complication and infection reductions.
Rai et al^23^	Wound healing categories: normal/healthy scar after suture removal, delayed wound healing, and infected	Preoperative nutritional status was among several factors governing postoperative wound healing. Preoperative malnourishment as assessed by the low levels of serum albumin, serum transferrin, and TLC did not necessarily lead to delayed wound healing. Although several authors found high incidences of delayed wound healing and wound infection in malnourished patients, there were none in this study. The risk imposed by malnutrition on wound healing can be modified by surgeon-dependent factors.
Savio et al^24^	Length of stay	Preoperative serum albumin was the only preoperative serum test associated with length of stay. Albumin was inversely related with length of stay.
Walls et al^25^	Mortality, superficial incisional SSI, and other	Hypoalbuminemia was a notable risk factor for mortality and major morbidity among THA patients, whereas morbid obesity was only associated with an increased risk of superficial SSI.
Yi et al^26^	Septic revision and aseptic revision	The presence of one or more laboratory parameters suggestive of malnutrition (ie, low albumin, low transferrin, and low lymphocyte count), although common in both normal weight and overweight patients, was independently associated with both chronic PJI and the development of an acute postoperative infection after an aseptic revision arthroplasty.

ACS-NSQIP = the American College of Surgeons National Surgical Quality Improvement Program; OR = odds ratio; PJI = periprosthetic joint infection; SSI = surgical site infection; THA = total hip arthroplasty; TLC = total lymphocyte count.

Table 2 is a continuation of Table 1 and lists the outcomes measured in and conclusions derived from each of the studies.

**Table 3:** RR of Postoperative Complications of Albumin Levels

Study	RR	Lower Limit CI	Upper Limit CI	% Weight
Kim et al^16^	1.724	1.038	2.861	13.05
Walls et al^25^	2.720	1.901	3.890	15.59
Courtney et al^11^	3.389	2.181	5.266	14.17
Kamath et al^6^	1.321	1.232	1.416	19.16
Bohl et al^10^	2.165	1.329	3.526	13.37
Nelson et al^4^	1.657	1.191	2.304	16.06
Kamath et al^15^	1.279	0.566	2.894	8.60
D + L pooled RR^a^ (*P*-value: < 0.001)	1.933	1.401	2.665	100.00

CI = confidence interval; RR = relative risk

**Figure 1:**
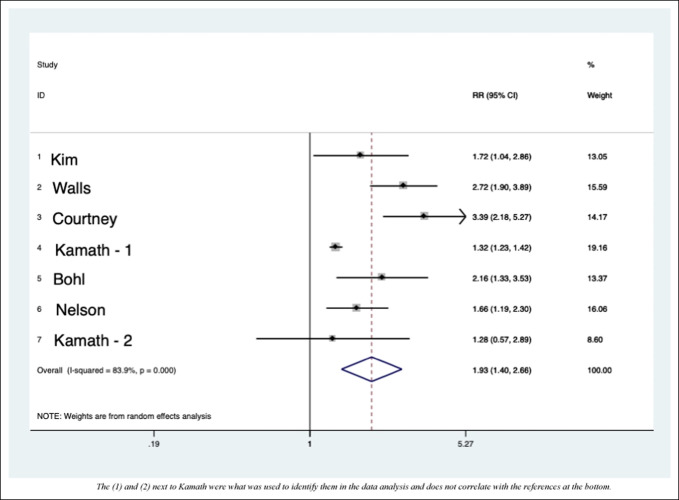
Graph showing the forest plot for the role of malnutrition in albumin articles based on studies with enough subjects. The (1) and (2) next to Kamath were what was used to identify them in the data analysis and does not correlate with the references at the bottom.

Table 3 is a numerical representation of Figure 1.

a Pooled RR using DerSimonian and Laird Random effects model.

**Table 4:** Biostatistical Numbers Used to Decide Albumin RR

Author	Normal Albumin Total	Normal Albumin Cases	Normal Albumin No cases	Low Albumin Total	Low Albumin Cases	Low Albumin No Cases	Explanation of Where Numbers Come From^a^
Kim et al^16^	839	29	810	470	28	442	AKI
Walls et al^25^	23,116	250	22,866	1122	33	1089	Any major complication
Courtney et al^11^	587	48	539	83	23	60	All complications^b^
Kamath et al^6^	3838	1740	2098	713	427	286	Any complication
Bohl et al^10^	3353	69	3284	449	20	429	PJI after revision for aseptic indication^c^
Nelson et al^4^	35,573	506	35,067	1570	37	1533	Any major complication
Kamath et al^15^	913	27	886	185	7	178	Unplanned ICU admission

ICU = intensive care unit; PJI = periprosthetic joint infection; RR = relative risk, AKI = acute kidney injury

a Where in the articles the data were retrieved from.

b Added all complications in Table 3, which is the addition of all patients from Table 2.

c The complication is the number of patients having a PJI after revision for an aseptic indication. Amount of albumin w/aseptic revisions = 3802 in Figure 1, which separates low albumin from norm albumin. The amount of normal albumin and low albumin w/complications is written in the last paragraph of the results section and is graphed out in Figure 3 of Bohl et al.^10^

We used a similar approach in the second part of the study, substituting the keywords “transferrin” and “total lymphocyte count.” There were 125 relevant article titles in PubMed, 108 relevant article titles in EMBASE, and three in Cochrane Library, totaling 236 relevant titles based on the question of interest. The same independent reviewers reviewed the titles, which yielded 66 relevant article abstracts. Of these 66, 54 full-text articles were selected after abstract evaluation. Including duplicates, 29 unique full-text articles were chosen for the final systematic review. On retrieval of the articles, we reviewed the following variables: primary outcome, secondary outcome, transferrin cutoff level used to define malnutrition, albumin cutoff level used to define hypoalbuminemia, TLC cutoff level used to define malnutrition, patient source, sample size, study design, and the number of cases and noncases among patients classified as normal nutrition and malnutrition. The resulting studies are listed in Tables 5–9.

**Table 5:** Demographics of Transferrin Articles With Enough Subjects to Perform Pooled Analysis

Author	Study Type	Patient Population	Transferrin Cutoff	Number Low Transferrin	Number Normal Transferrin	Low Transferrin or Albumin	Normal Transferrin and Albumin
Roche et al^27^	Retrospective	TKA	200	622	2339	N/A	N/A
Huang et al^14^	Prospective	THA and TKA	200	N/A	N/A	184	1977

**Table 6:** Outcomes and Conclusions of Transferrin Studies

Authors	Outcomes	Conclusions
Roche et al^27^	Postoperative infection, wound complications, concomitant infection with wound complications, and infection after wound complications	Patients with lower values of nutritional markers had higher incidences, and hence, odds ratios of complications.
Huang et al^14^	Multiple complications and length of stay	Malnutrition had higher rates of postoperative complications and length of stay.

Table 6 is a continuation of Table 5 and describes the outcomes and conclusions derived from each of the studies.

**Table 7:** RR of Transferrin as Risk of Postoperative Complications

Authors	RR	95% CI Lower Limit	95% CI Upper Limit	Weighted Percentage
Roche et al^27^	1.630	1.318	2.015	52.66
Huang et al^14^	4.076	2.555	6.501	47.34
D + L pooled RR (*P*-value: < 0.001)	2.515	1.022	6.191	100.00

CI = confidence interval; RR = relative risk

**Figure 2:**
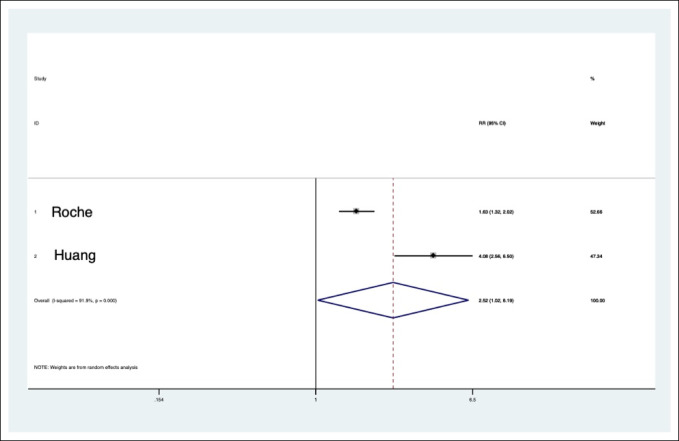
Graph showing the forest plot of transferrin articles.

Table 7 is a numerical representation of Figure 2. The information from Figure 2 and Table 7 were derived from the data in Table 8.

**Table 8:** Biostatistical Numbers Used to Determine RR of Transferrin

Author	Normal Transferrin Total	Normal Transferrin Cases	Normal Transferrin No Cases	Low Transferrin	Low Transferrin Cases	Low Transferrin No Cases	Explanation of Where Numbers Come From
Roche	2339	240	2099	622	104	518	Postoperative infection
Huang	1977	58	1919	184	22	162	Any complication

RR = relative risk

**Table 9:** Summary of Studies Using Transferrin or TLC as a Marker of Malnutrition

Definition of Nutrition	Definition of Malnutrition	Amount of Studies Showing Significance^a^	Total No. of Patients With Notable Results	Complications Which Reached Significance
All parameters within normal limits	Low albumin or transferrin or TLC	1^35^	463	Chronic septic failure and acute PJI complicating aseptic revision arthroplasty
Normal transferrin	Low transferrin	0^27^	3111	Wound complications^b^
All parameters within normal limits	Low albumin or transferrin	1^14^	2161	LOS, neurovascular, renal, hematoma/seroma, and any complications
Normal TLC	Low TLC	1^17^	101	Cost/charges, anesthesia time, surgical time, in-hospital costs, and LOS
All parameters within normal limits	Low albumin or TLC	2^2,19^	3169	Function score on the American Knee Society range of motion scale and^19^ low preoperative *S. albumin* associated with increased risk for SSIs^2^

PJI = periprosthetic joint infection; TLC = total lymphocyte count, LOS = length of stay

a The superscript next to the number refers to the article in which the data were extracted in each associated row. It is not an exponent.

b The single study showing significance did not list specific *P*-values, although it stated that a value of less than 0.05 was significant. It demonstrated, using percentages, that a low transferrin level led to an increased OR for wound complications. Although the study does not explicitly state that the data are significant, the words and the phrasing are highly suggestive of this.

The biostatistics were visualized using a random-effects forest plot. We compared data from all studies with sufficient data on patients with complications (ie, cases) and patients without complications (ie, noncases) among the two groups, normal albumin and hypoalbuminemia, to calculate a pooled RR combining the number of patients from all studies. A random-effects forest plot was also used to visualize information from the two studies with sufficient transferrin data.

## Results

When determining which studies had adequate detail regarding the numbers of cases and noncases, we found that seven studies had sufficient data for inclusion in the pooled analysis (Figure 1 and Tables 3 and 4). For each of the seven included studies, we chose only the complication of interest, “major complications” or “any complication,” if presented.

When malnutrition was defined as low transferrin, Roche et al^27^ identified an association with increased risk of postoperative infection (odds ratio [OR]: 1.87) and wound complications (OR: 1.9).

The study by Huang et al^14^ is the only study that describes malnutrition as low albumin or transferrin in the second half of the study. It found an increased length of stay (1.7 days), renal complications (OR: 2.85), and any complication (OR: 2.42).

The study by Yi et al^26^ is the only study that describes malnutrition as low albumin or transferrin or TLC in the second half of the study. It found an increased risk of chronic septic failure (OR: 2.13) and acute postoperative infection complicating an aseptic revision arthroplasty (OR: 5.9).

The study by Lavernia et al,^17^ the only study that describes malnutrition as low TLC in the second half of the study, found increased risks with increased cost/charges (*P*-value: 0.004), increased anesthesia time (*P*-value: 0.02), increase in surgical time (*P*-value: 0.002), increased in-hospital consults (*P*-value: 0.004), and increased length of stay (0.3 days).

The study by Morey et al,^19^ the only study that describes malnutrition as low albumin or TLC in the second half of the study, found the complication with increased risk was a decrease in range of motion in function score according to the American Knee Society scale (*P*-value: 0.009, amount of decrease in degrees: 1.5).

Meta-analysis of seven large-scale studies detailing the complications of albumin led to an all-cause RR increase of 1.93 when operating with hypoalbuminemia. This means that in the studies detailed enough to incorporate in this pooled analysis, operating on elective TJAs with low albumin is associated with a 93% increase in all measured complications. In the largest studies, analysis of transferrin levels for the most common complications revealed a RR increase of 2.52 when operating on patients with low transferrin levels. There were not enough subjects to do a biostatistical analysis in articles using TLC as the definition of malnutrition.

## Discussion

Most of the articles use 3.5 g/dL as the cutoff for hypoalbuminemia, with the range varying from 3.0 to 3.9 g/dL (Table 1). In the studies isolating only albumin, hypoalbuminemia is associated with increased risk for postoperative complications. Based on this systematic review, sufficient evidence is not available to make a statement regarding the risk for postoperative complications in patients with malnutrition as defined by low transferrin or low TLC.

As shown in Table 9, one article isolated TLC and one article isolated transferrin as markers for malnutrition, with a maximum study cohort size of 3111. The other articles included albumin as a component of their definitions and did not separate out the patients with only low albumin, which makes it difficult to identify whether, in their patient cohorts, low transferrin alone or low TLC alone led to increased risks of complications. Although there is not enough conclusive evidence to state that transferrin or TLC levels alone warrant delaying an elective TJA, that does not mean low transferrin or TLC are not present when there is low albumin. The articles studying albumin along with TLC or transferrin do not describe the relationship between them and treat them only as separate, isolated cohorts. Therefore, we are not able to state that low TLC or transferrin levels tend to accompany low albumin levels. In addition, only one^19^ of the 27 unique articles isolating albumin claims that albumin is not a reliable test of choice for identifying malnutrition. Despite the variability in methodologies, with certain studies selecting from the American College of Surgeons National Surgical Quality Improvement Program^3,5,10,12^ and certain studies^26^ being performed by a single surgeon on a team to limit variability, albumin seems to lead to an increased risk of postoperative complications, including mortality, unplanned readmissions, and increased length of stay.

## Conclusion

The focus is on the trends rather than absolutes. As shown in Table 1, whether the albumin cutoff for albumin was 3.0 g/dL, 3.5 g/dL, or 3.9 g/dL, the trend remains the same. Because low albumin before TJAs tends to increase complications, it is recommended to incorporate albumin levels in preoperative workups. Many patients with hip and knee arthritis undergo months of conservative management (eg, physical therapy and corticosteroid injections) before considering surgery, and it would be wise to optimize their nutritional status in this period to minimize the risk of perioperative complications. The physician should use these data to provide informed consent of the increased risk to patients planning to undergo TJAs with elevated malnutrition markers. Because this research is retrospective in nature, albumin should be studied prospectively in hypoalbuminemic and normoalbuminemic patients and their postoperative outcomes should be measured. Regarding transferrin and TLC, future research should help elucidate their predictive value and determine the value of preoperatively optimizing them and their effect in mitigating postoperative complications.
